# *Staphylococcus aureus* utilizes vimentin to internalize human keratinocytes

**DOI:** 10.3389/fcimb.2025.1543186

**Published:** 2025-02-21

**Authors:** Kyoungok Jang, Hangeun Kim, Dobin Choi, Soojin Jang, Dae-Kyun Chung

**Affiliations:** ^1^ Therapeutic Research Group, Antibacterial Resistance Laboratory, Institute Pasteur Korea, Seongnam-si, Gyeonggi-do, Republic of Korea; ^2^ Research and Development Center, Skin Biotechnology Center Co. Ltd., Yongin, Republic of Korea; ^3^ Graduate School of Biotechnology, Kyung Hee University, Yongin, Republic of Korea

**Keywords:** vimentin, *Staphylococcus aureus*, keratinocytes, HaCaT cells, skin infection, TLR signaling

## Abstract

**Introduction:**

Vimentin is an intermediate filamentous cytoskeletal protein involved in cell migration, adhesion, and division. Recent studies have demonstrated that several bacteria and viruses interact with vimentin to facilitate entry and trafficking within eukaryotic cells. However, the relationship between *Staphylococcus aureus* and vimentin remains unclear.

**Methods:**

In the current study, we elucidated vimentin expression mechanism in human keratinocytes infected with *S. aureus* using Western blot (WB), Flow cytometry, Immunofluorescence (IF) staining, utilizing neutralizing antibodies, and small interference (si) RNA, and a vimentin overexpression vector. The physical interaction between vimentin and *S. aureus* was shown by IF on cell surface, intra- and intercellular space.

**Results:**

HaCaT cells increased vimentin expression through physical interaction with live *S. aureus*, and not by heat-killed bacteria or bacterial culture supernatants. The Toll-like receptor (TLR) 2 signaling pathway, which includes interleukin 1 receptor-associated kinase (IRAK) and nuclear factor kappa B (NF-κB)/c-Jun N-terminal kinase (JNK) signaling activation, was involved in *S. aureus*-mediated vimentin expression. The vimentin protein induced by *S. aureus* was secreted extracellularly and bound to *S. aureus* in the culture media. The binding of vimentin to *S. aureus* accelerated the intracellular infection of HaCaT cells.

**Discussion:**

Thus, these experiments elucidated the mechanism of vimentin protein expression during *S. aureus* infection in human skin keratinocytes and revealed the role of vimentin in this process. These findings suggest that vimentin could serve as a potential target for the prevention or treatment of *S. aureus* infections.

## 1 Introduction

Vimentin is an intermediate filament protein whose expression is specifically increased in cells undergoing epithelial-mesenchymal transition (EMT). It is overexpressed in a variety of epithelial cancers, including lung cancer, breast cancer, gastrointestinal cancer, prostate cancer, malignant melanoma, and central nervous system cancers. The overexpression of vimentin in cancer cells is closely linked to tumor growth, invasion, and poor prognosis (Ivaska, 2011; Satelli and Li, 2011). Although vimentin was initially described in a limited number of physiological and pathophysiological contexts, recent findings have revealed that it plays diverse roles across a wide range of cellular and tissue functions. Additionally, vimentin is associated with various human diseases, including cataracts, cancer, Crohn’s disease, and HIV (Muller et al., 2009; Satelli and Li, 2011; Henderson et al., 2014). It is a crucial component of the cytoplasmic intermediate filaments (IFs) of astrocytes, which play vital roles in the organization of the central nervous system (CNS) and control many functions of the brain, spinal cord, and retina in both health and disease (Ridge et al., 2022).

On the other hand, vimentin plays a significant role in bacterial transport and the subsequent immune-inflammatory responses (Murli et al., 2001; Guignot and Servin, 2008; Su et al., 2019). When expressed on the cell surface, vimentin can exhibit both pro- and anti-bacterial properties, promoting bacterial invasion in certain contexts while limiting bacterial survival in others (Zou et al., 2006). Additionally, vimentin is secreted and found extracellularly, where it primarily regulates inflammation induced by bacterial infections (Mor-Vaknin et al., 2003). Vimentin has been shown to play important roles in virus attachment and entry of severe acute respiratory syndrome-related coronavirus (SARS-CoV), dengue and encephalitis viruses, among others. Moreover, the presence of vimentin in specific virus-targeted cells and its induction by proinflammatory cytokines and tissue damage contribute to its implication in viral infection (Ramos et al., 2020). Since vimentin is involved in bacterial and viral infections, and consequently induces inflammatory responses, which in severe cases can be life-threatening, developing drugs targeting vimentin could have the effect of controlling bacterial and viral infections (Miao et al., 2023). Although vimentin is thought to be involved in bacterial infection, evidence for infection mechanism through the interaction of vimentin and *S. aureus* in skin keratinocytes is unclear.

*S. aureus* is a species of gram-positive staphylococcus commonly found in the nasal cavity, respiratory tract, and on the skin. While it can lead to various infections, including skin infections, respiratory infections, sinusitis, and food poisoning, it may also exist as part of the normal bacterial flora without causing disease (Gehrke et al., 2023). On the skin, *S. aureus* is responsible for conditions such as folliculitis, boils, and impetigo, primarily through the infection of keratinocytes. Additionally, it can exacerbate atopic dermatitis and, although infrequently, may lead to necrotizing fasciitis as a result of severe soft tissue infections. Systemic infections caused by *S. aureus* can result in toxic shock syndrome. Despite being a component of the normal skin flora, *S. aureus* can pose significant health risks when the integrity of the skin barrier is compromised or when immune function is diminished. Furthermore, this bacterium frequently exhibits antibiotic resistance, complicating treatment efforts (David and Daum, 2017; Clebak and Malone, 2018; Geoghegan et al., 2018).

*S. aureus* can survive within human skin keratinocytes at concentrations 20 times higher than the standard minimal inhibitory concentration of commonly utilized anti-staphylococcal antibiotics, including flucloxacillin, teicoplanin, clindamycin, and linezolid, with the exception of rifampicin. Consequently, the internalization of *S. aureus* by human skin keratinocytes enables the bacteria to evade elimination by the majority of anti-staphylococcal antibiotics. This underscores the necessity for antimicrobial strategies that incorporate combinations of antibiotics capable of effectively penetrating animal cells to treat *S. aureus* infections (Al Kindi et al., 2019; Ngo et al., 2022).

Although cell surface receptors, such as fibronectin and integrins, are known to play a role in the intracellular infection of *S. aureus*, the function of vimentin has not yet been reported. Therefore, elucidating the role of vimentin in the infection of keratinocytes by *S. aureus* may enhance our understanding of immune evasion strategies and the pathogenesis of skin commensals, including *S. aureus*. The human skin serves as a primary defense mechanism and is frequently exposed to various pathogens, including *S. aureus*. Abnormal skin conditions, such as atopic dermatitis, create an environment conducive to *S. aureus* infection, allowing the bacteria to penetrate the stratum corneum, where it can proliferate and disseminate. Consequently, this study aims to elucidate the role of vimentin in the infection of keratinocytes by *S. aureus*, as well as the expression mechanisms triggered by the interaction between TLR2 present on the surface of HaCaT cells and *S. aureus*.

## 2 Results

### 2.1 *S. aureus* increased vimentin expression in HaCaT cells

To analyze gene variation in *S. aureus*-infected HaCaT cells, transcriptome analysis was performed (**Supplementary Figure 1**). Four genes—vimentin, SERPINE1, SPN, and PRELP—known to have a strong correlation with *S. aureus* infection, were selected from those whose expression increased more than fivefold in response to *S. aureus* in the quantitative RNA sequencing. In the following experiment, real-time PCR demonstrated that vimentin mRNA levels increased in a time-dependent manner, reaching approximately a nine-fold increase at 6 h post-infection, while the expression pattern of the other genes were atypical. Given that vimentin plays a crucial role in intracellular infections caused by viruses and bacteria (Ramos et al., 2020), we chose to further investigate vimentin, which was shown to be statistically and dose-dependently elevated by *S. aureus*, to evaluate its impact on *S. aureus* infection in HaCaT cells. Vimentin expression was observed not only in HaCaT cells, which are keratinocytes of the skin, but also in colonic epithelial cells. We observed that vimentin expression increased following *S. aureus* infection in HT-29, HCT-116, and CT-26 cells (**Supplementary Figure 2**). While vimentin demonstrates a response to *S. aureus* infection in various cell types, this study specifically focused on elucidating its expression mechanism in skin keratinocytes.

**Figure 1:**
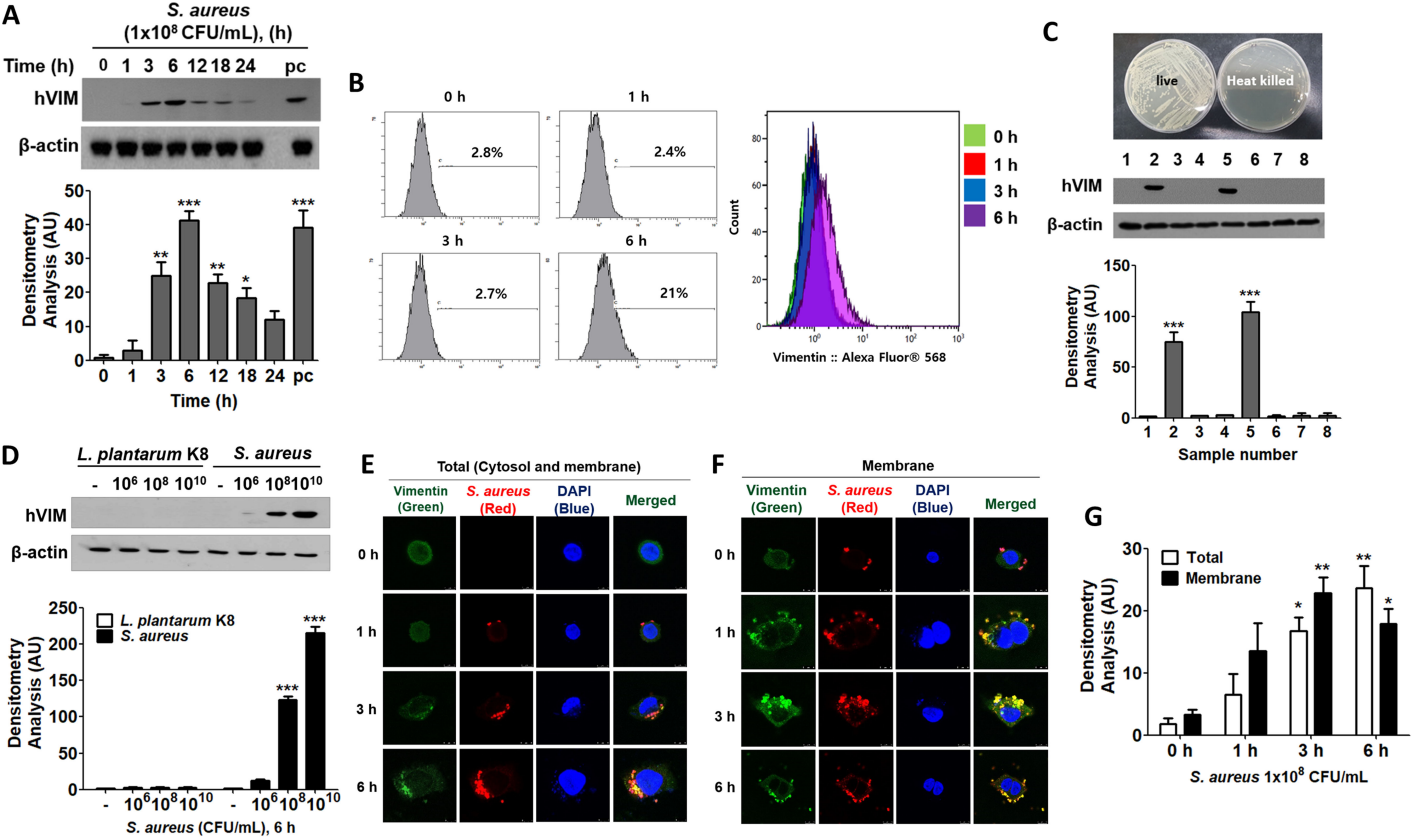
Vimentin expression in HaCaT cells treated with *S. aureus*. **(A)** HaCaT cells were treated with 1x10^8^ CFU/mL of *S. aureus* for the indicated times and cells were washed, lysed, and the protein levels were analyzed using Western blot (WB). **(B)** The cell surface expression of vimentin was assessed through fluorescence-activated cell sorting (FACS) analysis after treatment with 1x10^8^ CFU/mL of *S. aureus* for the indicated times. **(C)** HaCaT cells were treated with live and heat-killed *S. aureus* (1 x 10^8^ CFU/mL) and culture medium for 6 h. A transwell chamber was employed to separate the HaCaT cells from *S. aureus*. Following the harvesting of HaCaT cells, vimentin protein expression was evaluated using WB. Lane 1: Untreated; lane 2: Treatment with live *S. aureus*; lane 3: Treatment with heat-killed *S. aureus*; lane 4: Separate culture of *S. aureus* and HaCaT cells in a transwell chamber; lane 5: Non-isolated co-culture of *S. aureus* and HaCaT cells in a transwell chamber; lane 6: Treated with brain heart infusion (BHI) medium; lane 7: Treatment with culture supernatant from *S. aureus* cultured in BHI; lane 8: Treatment with culture supernatant from HaCaT cells treated with *S. aureus*. **(D)** HaCaT cells were treated with the specified doses of *L. plantarum* K8 and *S. aureus* for 6 h, after which intracellular vimentin was analyzed by WB. **(E, F)** Immunofluorescence staining was conducted on *S. aureus*-infected HaCaT cells. Cells were permeabilized prior to staining to assess cytoplasmic vimentin expression **(E)**, while intact cells were stained to evaluate membrane-bound vimentin expression **(F)**. **(G)** Densitometric image analysis of fluorescence intensities in panels **(E, F)** was conducted using ImageJ software. For panels **(A, C, D)**, densitometric image analysis of band intensities was also performed using ImageJ software. The data are presented as the mean ± SD and were statistically analyzed using a one-way ANOVA followed by Tukey’s multiple comparison test (for panels **A, C, G**) and a two-way ANOVA (for panel **D**). Statistical significance was defined as *p < 0.05, **p < 0.01, and ***p < 0.001.

It appears that HaCaT cells do not express detectable levels of vimentin protein under normal conditions. However, upon infection with *S. aureus*, vimentin levels increased for up to 6 h before declining (**Figure 1A**, upper panel). The relative quantities of protein bands from the Western blot are displayed in the lower panel of **Figure 1A**. In contrast to intracellular expression, the cell surface expression of vimentin remained unchanged up to 3 h post-infection but increased by 21% after 6 h (**Figure 1B**). The expression of vimentin in HaCaT cells was mediated by live *S. aureus*, as neither heat-killed *S. aureus* nor soluble factors in the culture supernatants influenced vimentin expression (**Figure 1C**, left panel). In experiments utilizing a transwell chamber, separate incubation of *S. aureus* and HaCaT cells did not induce vimentin expression. In contrast, vimentin expression was induced in the co-culture of *S. aureus* and HaCaT cells within a non-isolated chamber. The relative quantities of protein bands obtained from Western blots are presented in the right panel of **Figure 1C**. These results suggest that vimentin expression necessitates physical interaction between live *S. aureus* and HaCaT cells, and that an intact cell surface factor on *S. aureus* may play a crucial role in its interaction with host cells.

Vimentin expression was differentially regulated in HaCaT cells treated with probiotics and pathogens. As shown in **Figure 1D**, *Lactiplantibacillus plantarum* K8 did not induce vimentin expression in HaCaT cells, whereas it increased in a dose-dependent manner in *S. aureus*-treated HaCaT cells (**Figure 1D**, upper panel). The relative quantities of protein bands are displayed in the lower panel. This finding suggests that probiotics and non-pathogenic bacteria do not stimulate vimentin expression in HaCaT cells, which may influence bacterial infection. Vimentin expression increased in both the cytoplasm and on the cell surface in a time-dependent manner following *S. aureus* infection (**Figures 1E, F**). The relative quantities of fluorescence intensities are illustrated in **Figure 1G**, clearly demonstrating that the expression of total vimentin (both cytosolic and membrane-bound) and the expression of membrane-bound vimentin increased in a time-dependent manner. Immunofluorescence (IF) images demonstrated that vimentin was associated with *S. aureus* in the cytoplasm and on the cell surface, indicating that the binding of *S. aureus* to vimentin may impact the intracellular infection of *S. aureus* in HaCaT cells.

### 2.2 *S. aureus* internalized into HaCaT cells via vimentin

**Figure 2:**
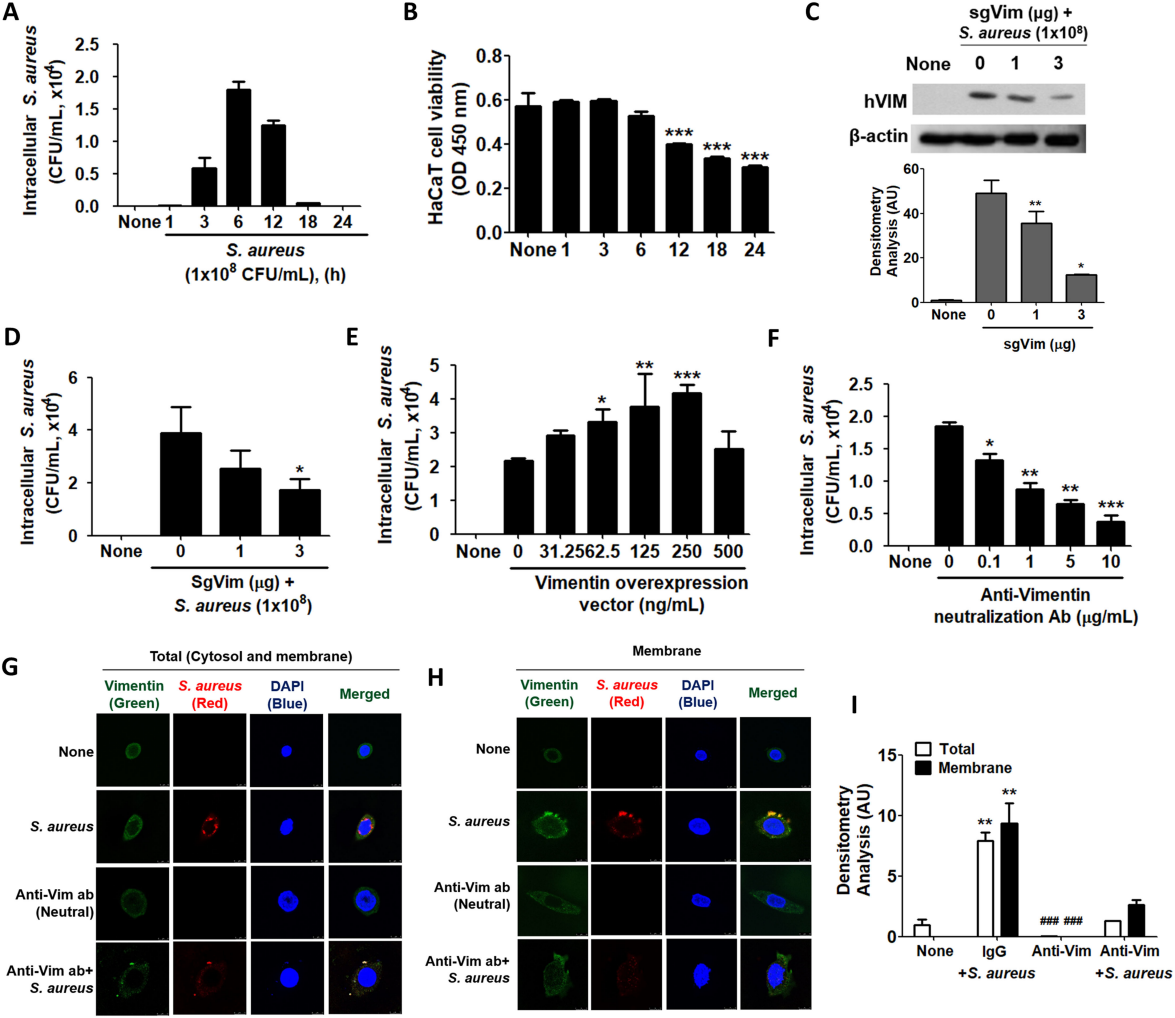
Intracellular infection of *S. aureus* using vimentin. **(A)** HaCaT cells were treated with 1x10^8^ CFU/mL of *S. aureus* for the indicated times, and cell lysates were plated on BHI agar to quantify colony-forming units (CFU). **(B)** Following infection with 1x10^8^ CFU/mL of *S. aureus* for the indicated time periods, a cell viability assay was conducted using WST-1 reagent. **(C, D)** Vimentin-specific sgRNA (sgVim) was transfected into HaCaT cells, which were subsequently treated with 1x10^8^ CFU/mL of *S. aureus* for 6 h Vimentin expression was evaluated using WB, and densitometric analysis of band intensities was conducted with ImageJ software. **(C)**. Intracellular *S. aureus* infection was evaluated by BHI agar plating of cell lysates **(D)**. **(E)** HaCaT cells were transiently transfected with the indicated dose of a vimentin overexpression vector and subsequently treated with 1x10^8^ CFU/mL of *S. aureus* for 6 h. The intracellular *S. aureus* infection was assessed by BHI agar plating of cell lysates. **(F)** HaCaT cells were pre-treated with control immunoglobulin (IgG) or with the indicated dose of an anti-vimentin neutralizing antibody for 30 min, followed by treatment with 1x10^8^ CFU/mL of *S. aureus* for 6 h. The intracellular *S. aureus* infection was evaluated through BHI agar plating of cell lysates. **(G, H)** HaCaT cells were pre-treated with control IgG or 10 μg/mL of an anti-vimentin neutralizing antibody for 30 min, followed by treatment with 1x10^8^ CFU/mL of *S. aureus* for 6 h. IF staining was performed to detect intracellular *S. aureus* **(G)** and cell surface-bound *S. aureus* **(H)**. **(I)** Densitometric image analysis of fluorescence intensities in images G and H was conducted using ImageJ software. Data were expressed as the mean ± SD and were statistically analyzed using a paired, one-tailed t-test **(A)** and one-way ANOVA followed by Tukey’s multiple comparison test in the other cases. *p < 0.05, **p < 0.01, and ***p < 0.001.

When HaCaT cells were treated with 1x10^8^ CFU/mL of *S. aureus* for the indicated times, intracellular *S. aureus* was increased up to 6 h and subsequently decreased (**Figure 2A**). This trend in *S. aureus* infection parallels the pattern of vimentin expression in HaCaT cells exposed to *S. aureus*, as shown in **Figure 1A**. HaCaT cell viability began to decline at 6 h post-infection with *S. aureus* and was significantly reduced by 12 h post-infection (**Figure 2B**). This decline in cell viability accounts for the decrease in intracellular CFU observed at 12 h post-infection in **Figure 2A**. Experiments utilizing CRISPR-Cas9 to knock down vimentin demonstrated that not only was cytoplasmic vimentin expression diminished, but intracellular *S. aureus* infection was also reduced (**Figures 2C, D**). The densitometric analysis of the Western blot presented in **Figure 2C** demonstrated a significant reduction in vimentin levels due to CRISPR-Cas9 treatment (**Figure 2C**, lower panel). Overexpression of vimentin in HaCaT cells via a transgenic vimentin expression vector resulted in a dose-dependent increase in intracellular *S. aureus* infection, with the exception of a high vector dose of 500 ng/mL (**Figure 2E**). Similar results were obtained in experiments employing an anti-vimentin neutralizing antibody. As shown in **Figure 2F**, intracellular *S. aureus* levels in HaCaT cells were significantly reduced in a dose-dependent manner with the neutralizing antibody. In IF images, intracellular *S. aureus* levels increased and were associated with vimentin protein, whereas *S. aureus* was barely detectable in cells treated with the anti-vimentin neutralizing antibody (**Figure 2G**). In control experiments, *S. aureus* bound to vimentin on the cell membrane; however, no binding between *S. aureus* and vimentin was observed in cells treated with the anti-vimentin neutralizing antibodies (**Figure 2H**). Notably, *S. aureus* was undetectable in the neutralizing antibody experiments, indicating that the anti-vimentin antibody effectively blocked *S. aureus* binding to cell surface vimentin (**Figure 2H**). Densitometric analysis of fluorescence intensities in the merged images presented in **Figures 2G, H** revealed consistent results: neutralizing antibodies against vimentin decreased both intracellular infection and membrane association of *S. aureus* when compared to control IgG treatment (**Figure 2I**).

Unlike *S. aureus*, probiotics such as *L. plantarum* and *Enterococcus faecium* rarely cause intracellular infections in HaCaT cells. When HaCaT cells were treated with 1x10^8^ CFU/mL of bacteria for 6 h, *L. plantarum* was infected 400 times less frequently, and *E. faecium* was infected approximately 50 times less frequently than *S. aureus* (**Supplementary Figure 3**). These probiotics did not induce vimentin expression in HaCaT cells, suggesting a close association between vimentin and the intracellular infection of bacteria. In contrast, *Escherichia coli* and *Shigella flexneri*, which are classified as pathogens, induced less vimentin expression in HaCaT cells compared to *S. aureus* and caused approximately 40 to 80 times less intracellular infection (**Supplementary Figure 4**). These results further imply a strong correlation between vimentin expression and infection in HaCaT cells.

### 2.3 Secreted vimentin binds to *S. aureus* and facilitates intracellular infection

**Figure 3:**
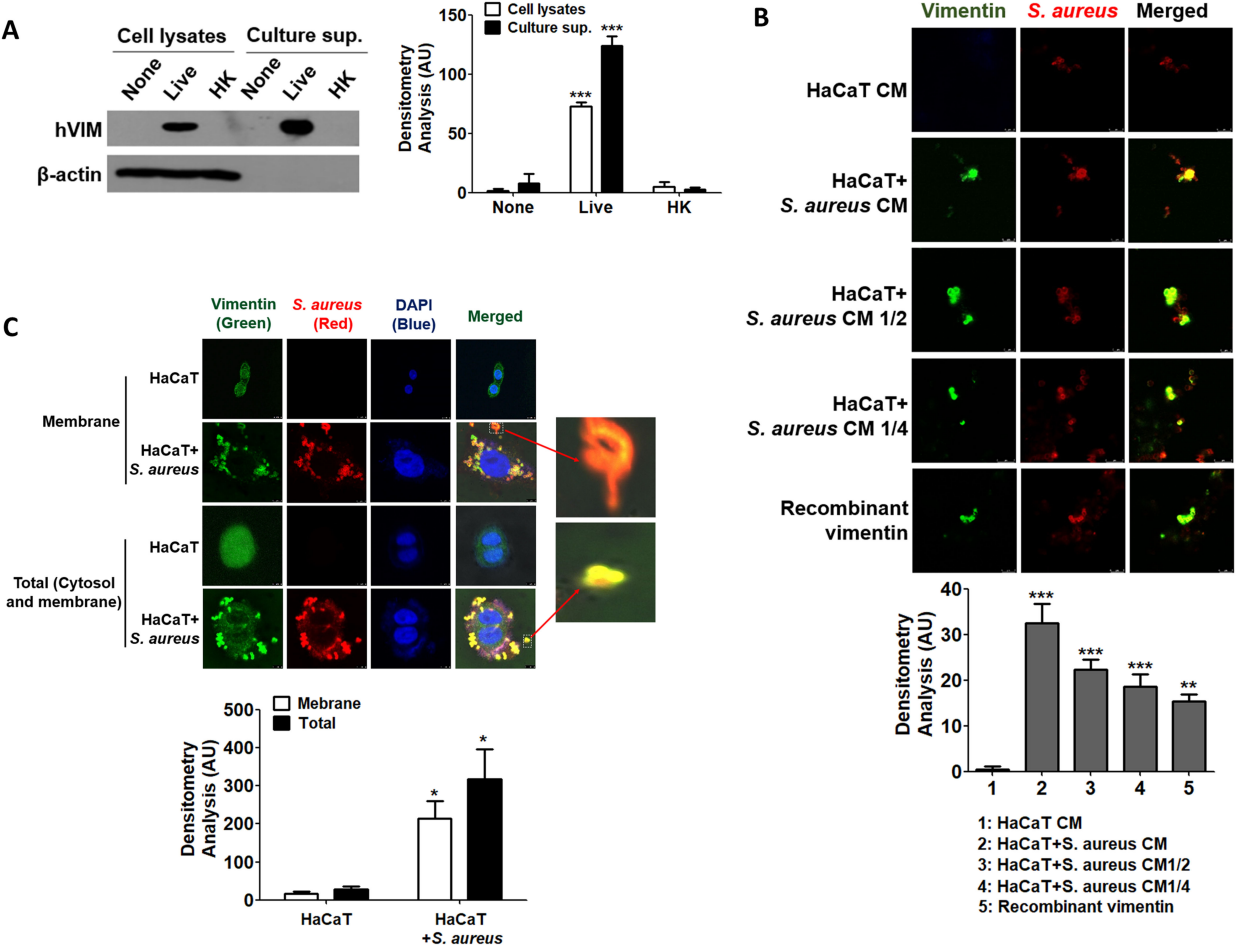
Binding of secreted vimentin to *S. aureus*. **(A)** HaCaT cells were treated with 1x10^8^ CFU/mL of live *S. aureus* or heat-killed *S. aureus* for 6 h, and vimentin was detected by WB in both cell lysates and culture supernatants. **(B)** CM were prepared from untreated HaCaT cells and from HaCaT cells treated with 1x10^8^ CFU/mL of *S. aureus* for 6 h. CM, diluted CMs, and recombinant vimentin were incubated with 2x10^6^ CFU/mL of live *S. aureus* for 1 h, followed by IF analysis. **(C)** HaCaT cells were treated with 1x10^8^ CFU/mL of *S. aureus* for 6 h, and IF was performed to observe the interaction between vimentin and *S. aureus* in the cytoplasm and on the cell surface. The binding of *S. aureus* to vimentin, which detached from HaCaT cells, is shown enlarged in the box. Densitometric image analysis of band intensities **(A)** and fluorescence intensities **(B, C)** was conducted using ImageJ software. The data are expressed as the mean ± SD and were statistically analyzed using a one-way ANOVA followed by Tukey’s multiple comparison test in the other instances. *p < 0.05 and ***p < 0.001.

It is known that vimentin is secreted into the extracellular space and plays a role in bacterial killing and the generation of oxidative metabolites in activated macrophages (Mor-Vaknin et al., 2003). In this study, we investigated whether vimentin levels increase in HaCaT cells treated with *S. aureus* and whether it is secreted extracellularly to interact with the bacteria. Notably, only live *S. aureus* induced vimentin expression in HaCaT cells, and vimentin was detected in the extracellular space (**Figure 3A**, left panel). The relative quantities of protein bands obtained from Western blots are shown in the right panel of **Figure 3A**. Vimentin expression was significantly increased both intracellularly and extracellularly by live *S. aureus*. To investigate the interaction between *S. aureus* and vimentin, IF analysis was performed. As shown in **Figure 3B**, when *S. aureus* was added to conditioned media (CM) derived from unstimulated HaCaT cells, only *S. aureus* was detected, while vimentin was absent. Conversely, when *S. aureus* was added to CM obtained from HaCaT cells previously treated with *S. aureus*, both vimentin and *S. aureus* were detected, and binding between vimentin and *S. aureus* was observed. The relative quantities of fluorescence intensity are displayed in the lower panel of **Figure 3B**, indicating that the intensity decreased proportionally with dilution. Similar results were obtained when *S. aureus* was added to diluted CMs or media containing recombinant vimentin. The binding of vimentin to *S. aureus* was observed in both the intracellular (cytoplasmic and membrane) regions and at the cell surface (**Figure 3C**). The relative quantities of fluorescence intensities are presented in the lower panel of **Figure 3C**, indicating that *S. aureus* infection enhances vimentin expression and its binding. Additionally, vimentin binding to *S. aureus* was observed at locations distant from the cell (see enlarged box), suggesting that vimentin may interact with *S. aureus* in the extracellular space rather than solely at the cell surface, potentially facilitating the intracellular infection of *S. aureus*.

### 2.4 TLR2 was primarily involved in the induction of vimentin in HaCaT cells treated with *S. aureus*

**Figure 4:**
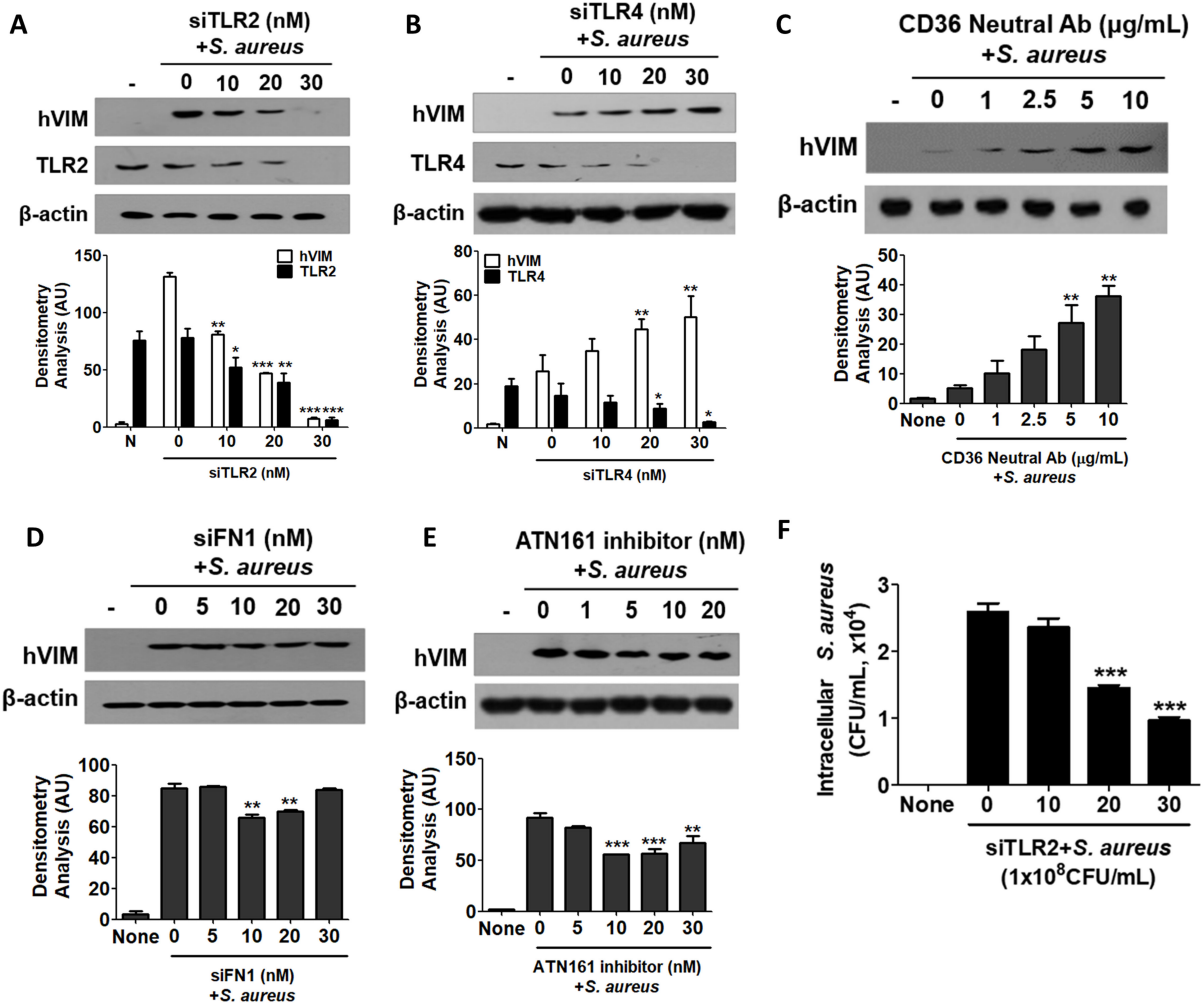
Cell surface receptors involved in signaling initiation. **(A-E)** HaCaT cells were pre-treated for 30 min with small interfering RNA targeting TLR2 **(A)**, TLR4 **(B)**, neutralizing antibodies against CD36 **(C)**, FN1 **(D)**, or an inhibitor of ATN161 **(E)**. Subsequently, the cells were exposed to 1 x 10^8^ CFU/mL of *S. aureus* for 6 h. Cell lysates were analyzed using Western blotting to assess intracellular vimentin expression. **(F)** HaCaT cells were transiently transfected with siTLR2 RNA and subsequently treated with 1x10^8^ CFU/mL of *S. aureus* for 6 h. Following lysis, the cells were plated onto BHI agar plates, and CFU were counted after overnight incubation. Densitometric image analysis of band intensities was conducted using ImageJ software **(A-E)**. The data are presented as the mean ± SD and were statistically analyzed using one-way ANOVA followed by Tukey’s multiple comparison test. A significance level of *p < 0.05, **p < 0.01, and ***p < 0.001 was observed.

*S. aureus* is known to interact with Toll-like receptors (TLRs), fibronectin (FN), and integrins (Kielian et al., 2005; Stenzel et al., 2008; Josse et al., 2017). We investigated which receptor is involved in the initiation of vimentin expression by *S. aureus* in HaCaT cells. First, small interfering RNA (siRNA) targeting TLR2 and TLR4 was applied, and a decrease in the expression of TLR2 and TLR4 was observed by WB (**Figures 4A, B**). siTLR2 RNA inhibited vimentin expression in a dose-dependent manner, whereas siTLR4 had no effect. Densitometric analysis of protein bands yielded similar results (Lower panels in **Figures 4A, B**). Vimentin expression was significantly decreased in TLR2 knockdown cells, while it remained significantly increased in TLR4 knockdown cells. Neutralizing antibody against CD36 also showed no inhibitory effect on vimentin expression in HaCaT cells treated with *S. aureus* (**Figure 4C**). siRNA targeting fibronectin 1 (FN1) significantly reduced vimentin expression in cells treated with 10 nM and 20 nM of FN1 siRNA (**Figure 4D**). Additionally, vimentin expression was decreased by the ATN161 inhibitor, an antagonist of integrin α5β1, suggesting that α5β1 may play a role in *S. aureus*-mediated vimentin expression (**Figure 4E**). The results of the densitometric analysis of protein bands are presented in the lower panel of each figure. Collectively, these findings indicate that TLR2 is primarily involved in vimentin expression in HaCaT cells treated with *S. aureus*, while fibronectin and integrin also contribute to vimentin expression. Notably, when HaCaT cells were transiently transfected with siTLR2 RNA, intracellular *S. aureus* infection was significantly reduced in a dose-dependent manner (**Figure 4F**).

### 2.5 NF-κB and JNK signaling pathways are implicated in the regulation of vimentin expression in HaCaT cells exposed to *S. aureus*

**Figure 5:**
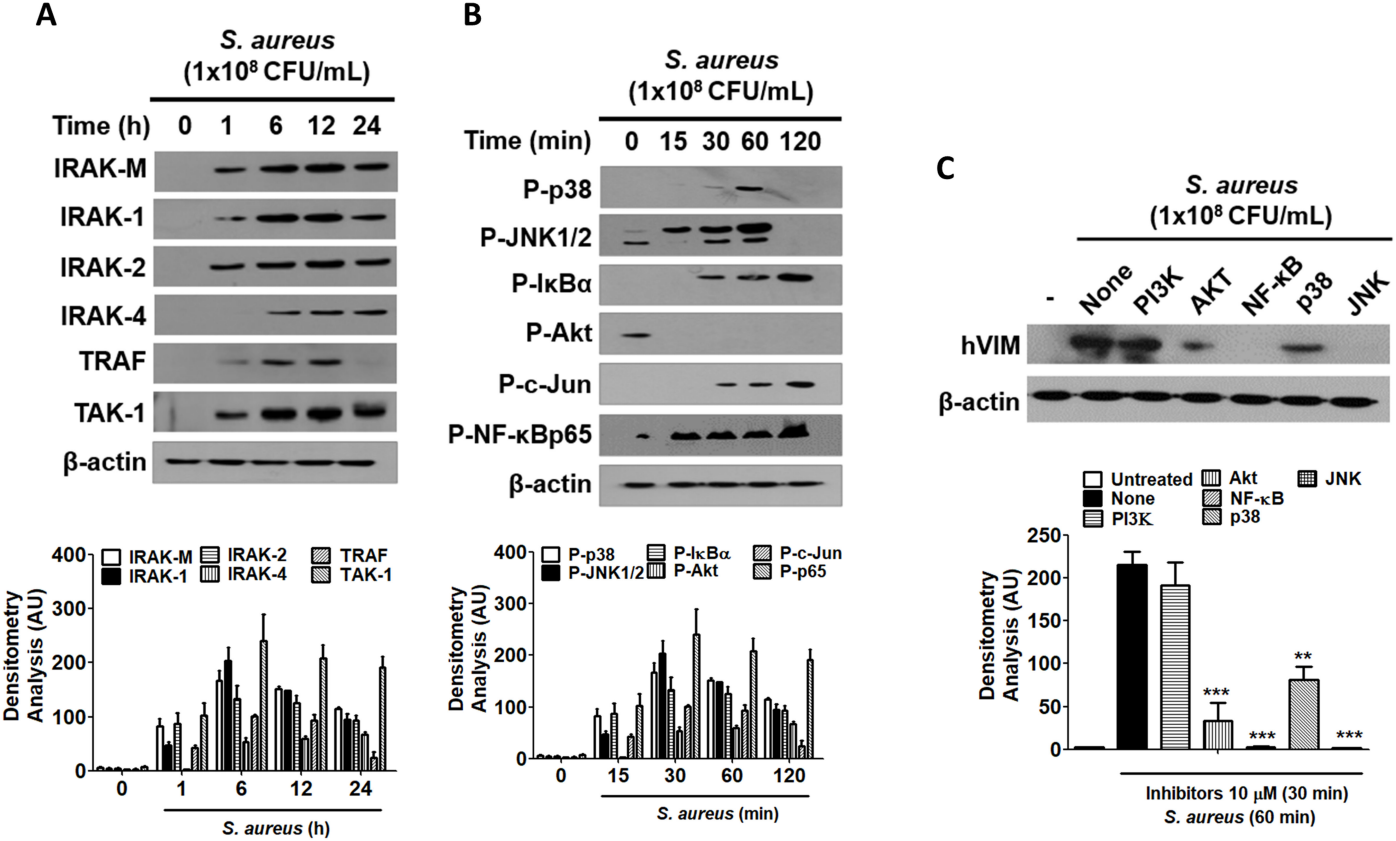
The role of NF-κB and JNK in vimentin expression. **(A, B)** HaCaT cells were treated with 1x10^8^ CFU/mL *S. aureus* for the indicated times. TLR2 associated protein levels were examined by WB **(A)** and the activation of signaling molecules were detected by WB **(B)**. **(C)** HaCaT cells were pre-treated with 10 μM inhibitors for 30 min, followed by exposure to 1 x 10^8^ CFU/mL *S. aureus* for 60 min. Vimentin expression was analyzed via WB using cell lysates. Densitometric image analysis of band intensities was conducted using ImageJ software and is presented in the lower panels (**A-C**). The data are presented as the mean ± SD and were statistically analyzed using one-way ANOVA followed by Tukey’s multiple comparison test. A significance level of **p < 0.01 and ***p < 0.001 was observed.

Signaling initiation through TLR2 recruits interleukin-1 receptor-associated kinase (IRAK) proteins, with IRAK4 forming a heterodimer with either IRAK1 or IRAK2 (Pereira and Gazzinelli, 2023). The expression of IRAK family proteins was found to increase in a time-dependent manner in HaCaT cells treated with 1x10^8^ CFU/mL of *S. aureus* (**Figure 5A**). Additionally, the protein levels of downstream signaling molecules, such as tumor necrosis factor receptor-associated factor 6 (TRAF6) and transforming growth factor-β-activated kinase 1 (TAK1), also exhibited a time-dependent increase, indicating that the TLR2 signaling pathway was activated by *S. aureus*. Notably, the protein expression of IRAK-M was also elevated, which may play a role in inhibiting *S. aureus*-mediated signaling initiated at TLR2 at some stage. The TLR2-mediated signaling pathway initiated by *S. aureus* led to the activation of p38, c-Jun N-terminal kinases (JNK)1/2, NF-kappa-B inhibitor alpha (IκBα), and transcription factors such as c-Jun and nuclear factor kappa B (NF-κB) (**Figure 5B**). The results of the densitometric analysis of protein bands are presented in the lower panels of each figure. Furthermore, when HaCaT cells were treated with inhibitors targeting specific signaling molecules prior to *S. aureus* exposure, the inhibitors for NF-κB and JNK resulted in a reduction of vimentin expression, suggesting that these signaling pathways are primarily implicated in *S. aureus*-mediated vimentin expression in HaCaT cells (**Figure 5C**, upper panel). Densitometric analysis indicated that the Akt and p38 signaling pathways were also implicated in vimentin expression (**Figure 5C**, lower panel).

## 3 Discussion

Through this experiment, we discovered that vimentin, which is expressed following the binding of *S. aureus* to TLR2 on skin keratinocytes, facilitates the internalization of *S. aureus* into HaCaT cells. Notably, heat-killed *S. aureus* did not induce vimentin expression in HaCaT cells, nor did it result in internalization. Only live *S. aureus* was found to induce vimentin expression in HaCaT cells and influence the internalization process. This observation suggests that a component on the surface of *S. aureus* interacts with TLR2, and this protein appears to be particularly sensitive to heat. Generally, ligands that bind to TLR2 in bacteria include lipoteichoic acid (LTA), lipoproteins, and peptidoglycan (Fournier, 2013). Among these, LTA is relatively heat-stable (Grunfeld et al., 1999). Conversely, peptidoglycan and lipoproteins are susceptible to heat, leading to the expectation that these substances would bind to TLR2 in HaCaT cells and induce vimentin expression (Cebrián et al., 2017; Jiang et al., 2023). Previous research has indicated that the transcription factors AP-1 and NF-kB are crucial for vimentin expression (Paulin et al., 2022). In this study, we demonstrated that the JNK and NF-kB signaling pathways are significant in vimentin expression in HaCaT cells treated with *S. aureus*, as evidenced by experiments utilizing specific inhibitors. The activation of the JNK and NF-kB pathways occurs via IRAK proteins following the binding of *S. aureus* to TLR2. Vimentin expressed in response to *S. aureus* was detected not only in the cytoplasm of skin keratinocytes but was also secreted onto the cell surface and into the culture medium. Intracytoplasmic vimentin contributes to the mechanical integrity of cells and aids in the localization of intracellular components (Guo et al., 2013). Cell surface vimentin may function as a receptor or ligand for endogenous proteins, such as von Willebrand factor (vWF), as well as for exogenous proteins, including those derived from bacteria and viruses (Paulin et al., 2022). However, the role of vimentin that is secreted into the intercellular space, particularly in the context of bacterial intracellular infection, remains poorly understood. In this study, we demonstrated that vimentin secreted into the intercellular space binds to *S. aureus* and facilitates its entry into the cell. These findings contribute to a better understanding of the role of secreted vimentin in bacterial infection.

Secreted vimentin has been shown to bind to *S. aureus*, facilitating intracellular infection. Notably, findings from our experiments suggest a correlation between vimentin expression and *S. aureus* infection. Specifically, probiotics did not induce vimentin expression, which consequently resulted in a lack of intracellular internalization. In contrast, *E. faecium*, *E. coli*, and *S. flexneri* exhibited significantly lower levels of intracellular internalization compared to *S. aureus*, and these bacteria also did not induce high levels of vimentin expression relative to *S. aureus*. This indicates a strong relationship between vimentin expression and the intracellular infection process of *S. aureus*. Probiotics, such as *L. plantarum* K8, did not promote vimentin expression, leading to the conclusion that intracellular infection did not occur in these cases. Conversely, while *E. coli* and *S. flexneri* were not expressed in this experiment, they demonstrated higher rates of intracellular internalization into HaCaT cells compared to probiotics. This difference can be attributed to the ability of gram-negative pathogens to utilize various infection routes, as noted in previous studies (Gophna et al., 2001; Schroeder and Hilbi, 2008). However, some literature indicates that *E. coli* can enhance vimentin expression and interact with it during the process of intracellular internalization (Chi et al., 2010). The absence of this phenomenon in our experiment may be attributed to the specific characteristics of the HaCaT cells utilized or the possibility that vimentin expression in *S. aureus* was sufficiently robust to remain relatively undetectable.

Current knowledge identifies several factors that mediate the binding of pathogens to vimentin, including IbeA and MBP-1. Additionally, various bacterial factors, such as SptP, SpyA, CPAF, and AptA, have been reported to interfere with vimentin’s function, potentially through mechanisms involving proteolysis or post-translational modifications (Chi et al., 2010; Humphreys et al., 2009; Icenogle et al., 2012; Snavely et al., 2014; Babrak et al., 2015; Zhao et al., 2024). To date, there has been no investigation into the relationship between vimentin and the intracellular infection of *S. aureus*. In the present study, we confirmed the binding of vimentin to *S. aureus* through IF and neutralizing antibody assays. However, the specific factor of *S. aureus* that interacts with vimentin remains unidentified. The attachment and internalization of *S. aureus* may be modulated by Staphylococcal protein A (spa) and coagulation factor B (clfB), which are produced by the regulatory factor SpoVG, a known general regulatory element in *S. aureus* (Zhu et al., 2019). The pathogenesis of invasive *S. aureus* infections is attributed to various virulence factors, including protein A and alpha-hemolysin (Hla), which engage host signaling pathways to induce pathological effects. *S. aureus* exploits the inflammatory predisposition of human keratinocytes to trigger pyroptosis, a form of inflammatory cell death that is dependent on caspase 1, which is essential for the bacterium’s ability to penetrate the keratinocyte barrier (Soong et al., 2012). The infection of skin keratinocytes by *S. aureus* can involve several mechanisms, including the utilization of sphingosine 1-phosphate and its receptor, integrin-linked kinase, Rac1, the Chemerin-CMKLR1 axis, fibronectin-binding protein, and multiple high-affinity fibronectin-binding repeats within fibronectin-binding protein A (Edwards et al., 2011; Bur et al., 2013; Nguyen et al., 2018; Igawa et al., 2019; Chen et al., 2024). From the perspective of a host cell, TLR2 and lipoprotein-like lipoproteins have been implicated in the infection process of *S. aureus* (Sayedyahossein et al., 2015). In addition, actin filaments, microtubules, receptor-mediated endocytosis, and protein tyrosine kinases are critical for the uptake of *S. aureus*. Furthermore, fibronectin-binding protein and β1-integrin have been identified as essential cell surface molecules that facilitate the internalization of *S. aureus* by non-phagocytic cells (Alexander and Hudson, 2001). Future research should focus on a comprehensive examination of the interactions between the surface proteins of *S. aureus* and the receptors present on the surface of eukaryotic cells.

It can be challenging to recognize bacterial infections as a significant contributor to cancer. Nevertheless, studies have demonstrated two primary mechanisms by which bacteria are associated with cancer: the production of carcinogenic metabolites and the induction of chronic inflammation. For example, certain species of Bacteroides can generate phenocarpentaene, a potent mutagen, in substantial quantities under laboratory conditions. *Helicobacter pylori* has a lifelong propensity to induce inflammation and is epidemiologically linked to adenocarcinoma of the distal stomach (Parsonnet, 1995). *H. pylori* infection is recognized as a contributing factor to stomach cancer, while persistent chlamydial infection is a risk factor for the development of cervical cancer, particularly in patients who are concurrently infected with human papillomavirus (HPV) (Yusuf et al., 2023). Among survivors of *S. aureus* bacteremia (SAB), the risk of developing primary cancer was found to be 65% higher than in a randomized control group, suggesting that susceptibility to infectious diseases may serve as a marker of immunodeficiency related to cancer (Gotland et al., 2020). Indeed, chronic *S. aureus* infection has been associated with an increased risk of certain cancers, including skin and oral cancers. Chronic inflammation resulting from persistent *S. aureus* infection may lead to DNA damage, disrupt cell signaling pathways, and create an immunosuppressive microenvironment that promotes cancer progression (Odunitan et al., 2024). Vimentin is known to be overexpressed in various epithelial malignancies, including breast, gastrointestinal, prostate, central nervous system, lung cancers, and malignant melanoma (Satelli and Li, 2011). For example, patients diagnosed with vimentin-positive gastric cancer exhibit a significantly poorer prognosis compared to those with vimentin-negative gastric cancer (Fuyuhiro et al., 2010). The presence of vimentin in cancer cells, particularly its secretion into the extracellular environment, is believed to facilitate infections by *S. aureus*. Given vimentin’s role in both the metastasis of cancer cells and susceptibility to bacterial infections, exploring the modulation of vimentin may be a viable strategy for achieving both anticancer and antibacterial therapeutic effects.

In conclusion, *S. aureus* enhances the expression of vimentin protein via TLR2 signaling in keratinocytes. Vimentin is secreted intracellularly, presented on the cell surface, and released into the extracellular medium, where it binds to *S. aureus*, facilitating the internalization of the bacteria into HaCaT cells. Infected *S. aureus* has the ability to evade the host immune response, allowing for its proliferation and subsequent release. Given the critical role of vimentin in the life cycle of *S. aureus*, there is potential for the development of therapeutics that target vimentin to manage intracellular infections caused by this pathogen.

## 4 Materials and methods

### 4.1 Reagents

Neutralizing antibodies, including anti-TLR2 (Cat# MA5-14112) and anti-CD36 (Cat# MA5-11883), were purchased from Invitrogen (MA, USA). siRNAs targeting TLR2 (FlexiTube Genesolution GS7097), TLR4 (GS7044), and FN1 (GS2335) were prepared from Qiagen (Hilden, Germany). ATN-161, an integrin α5β1 receptor antagonist (Cat# 6058), was purchased from R&D Systems (Minneapolis, MN, USA). Inhibitors targeting PI3K (S1109), Akt (S1113), NF-κB (S4902), p38 (S1076), and JNK (S1460) were purchased from Selleck Chemicals LLC (Houston, TX, USA). The primary antibodies used in Western blot (WB) analysis included IRAK-M (Cat# 4369), IRAK-1 (Cat# 4504), IRAK-2 (Cat# 4367), IRAK-4 (Cat# 4363), TRAF (Cat# 8028), TAK-1 (Cat# 4505), phospho-p38 (Cat# 4511), phospho-JNK (Cat# 9251), phospho-IκBα (Cat# 2859), phospho-Akt (Cat# 4060), phospho-c-Jun (Cat# 3270), and phospho-NF-κB p65 (Cat# 3033). These antibodies were acquired from Cell Signaling Technology (Danvers, MA, USA). Rabbit anti-vimentin antibody was purchased from Abcam (ERP3776, Cambridge, UK).

### 4.2 Cell culture

The human immortalized epidermal keratinocyte cell line HaCaT was maintained in Dulbecco’s Modified Eagle’s Medium (DMEM) supplemented with 10% heat-inactivated fetal bovine serum (FBS), 2 mM L-glutamine, 100 U/ml penicillin, and 100 μg/ml streptomycin. These cells were cultured at 37°C in a humidified incubator with 5% CO_2_, and the medium was changed every 3 to 4 days. To investigate the induction of vimentin expression by *S. aureus* in HaCaT cells, live and heat-killed *S. aureus* were treated to HaCaT cells at specified concentrations and durations. Isolation culture was conducted using a transwell chamber (Corning^®^ Transwell^®^ 6-well plates, CLS3428, Sigma-Aldrich).

### 4.3 Bacteria preparation

*Lactiplantibacillus plantarum* K8 (KCTC10887BP) was cultured in 1 L of MRS broth (BD Biosciences, USA) at 37°C overnight. The cells were then harvested by centrifugation at 8000× g for 8 min. *L. plantarum* K8 was washed three times with Dulbecco’s phosphate-buffered saline (DPBS) and resuspended in DMEM to achieve the desired concentration. *S. aureus* (ATCC 29523), obtained from the American Type Culture Collection, was inoculated in Brain Heart Infusion (BHI) broth (BD Difco, NJ, USA) and incubated at 37°C for 16 to 18 h in a shaking incubator maintained at 220 rpm. After 24 h, the *S. aureus* culture was transferred to fresh BHI medium and cultured until the exponential phase (OD600 = 1.0) was reached. The bacteria were harvested by centrifugation at 4,000 x g for 10 min and washed three times with DPBS. The resulting pellets were resuspended in DMEM to achieve the desired concentration. To prepare heat-killed bacteria, a separate *S. aureus* culture was subjected to the same conditions and then heat-treated at 90°C for 30 min, with vortexing at 5-min intervals during the heat treatment.

*Escherichia coli* and *Shigella flexneri* were cultured overnight in a shaking incubator at 37°C using Luria Broth (LB) medium. Each bacterium was diluted 1:100 into 100 mL of fresh LB broth and subcultured at 37°C for 6 h. The cultured bacteria were harvested by centrifugation at 4,000 x g for 10 min and washed three times with DPBS. The resulting pellets were resuspended in DMEM to achieve the desired concentration.

### 4.4 Construction of CRISPR/Cas9-Based knockdown of vimentin

The construction of the GRISPR/Cas9 vector that targeted vimentin was carried out according to the protocol provided by the vector vendor Toolgen (Seoul, Korea). The CRISPR guide RNA (gRNA) for human vimentin (TCC TAC CGC AGG ATG TTC GGC GG) was designed using the gRNA design tool from GenScript (NJ, USA) and subsequently cloned into the pRGEN-Cas9-CMV expression vector (Bioneer, Daejeon, Korea). Synthesized gRNA vectors (1 μg, 2 μg, 3 μg) and Lipofectamine were mixed with Opti-MEM and added to HaCaT cells (1 x 10^5^ cells/mL) that had been stabilized through overnight culture. Subsequently, 1 μg of donor DNA was incorporated into the mixture and allowed to react at room temperature for 30 min. The resulting gRNA/DNA mixture was then transfected into HaCaT cells and incubated at 37°C for 42 h. Following this incubation period, *S. aureus* was introduced at a multiplicity of infection (MOI) of 200. The cells were harvested after 6 h and the reduction in vimentin levels was confirmed via Western blot analysis. After lysing the HaCaT cells with a hypertonic buffer, the cells were plated on BHI plates and the *S. aureus* colonies that had entered the cells were counted.

### 4.5 HaCaT cell infection with *S. aureus*

HaCaT cells were transfected with siRNA, pretreated with inhibitors, or left untreated before being infected with *S. aureus*. For siRNA transfection, HaCaT cells were seeded in 6-well plates (6 x 10^5^ cells/well) and transfected with indicated dose of vimentin siRNA using 6 μl of G-fectin (Genolution, Seoul, Korea). The cells were then incubated for 48 h in a humidified incubator at 37°C with a 5% CO_2_ atmosphere. For the inhibitor assays, HaCaT cells were pretreated with the indicated doses of inhibitors for 30 min prior to *S. aureus* infection. The indicated dose of *S. aureus* was added to HaCaT cell cultures and incubated at 37°C in a humidified 5% CO_2_ incubator for 6 h. After incubation, the cells were washed three times with DPBS and replaced with fresh DMEM medium containing gentamicin (100 μg/mL), followed by an additional incubation for 1 h to eliminate any remaining extracellular *S. aureus*. The cells were then washed three times with DPBS, and 1 mL of hypertonic buffer [20 mM Tris (pH 7.5), 5 mM MgCl2, 5 mM CaCl2, 1 mM DTT, 1 mM EDTA, 0.1% Triton X-100] was added and incubated at 4°C for 5 min to lyse the infected HaCaT cells. Cell lysates were spread on BHI agar plates, incubated at 37°C, and colonies were counted the following day.

### 4.6 Western blot analysis

HaCaT cells infected with *S. aureus* were lysed with 2X reducing sample buffer and separated by 10% (v/v) denaturing sodium dodecyl sulfate-polyacrylamide gel electrophoresis (SDS-PAGE) for 2 h at 80 V. Following migration, the proteins were transferred onto a polyvinylidene difluoride (PVDF) membrane (GE Healthcare, Chicago, IL, USA) for 1 h at 100V. The membrane was pre-incubated in blocking buffer [5% (w/v) non-fat dried milk in Tris-buffered saline with 0.05% (v/v) Tween-20 (TBS-T)] for 1 h at room temperature (RT). Subsequently, the membranes were incubated overnight at 4 ˚C with first antibodies. After washing the membranes three times with TBS-T (20 mM Tris-HCl, 150 mM NaCl, 0.05% Tween-20), they were incubated with horseradish peroxidase (HRP)-conjugated anti-rabbit IgG (ab205718, Abcam) secondary antibody for 2 h at RT. Following five washes with TBS-T, the protein bands were detected using ECL Select™ Western Blotting Detection Reagent (Cytiva, MA, USA) and exposed on X-ray film. *β*-actin (sc-47778, Santa Cruz Biotechnology, CA, USA) was used as an internal loading control.

### 4.7 Immunofluorescence

HaCaT cells (2 x 10^4^ cell/well) were seeded in glass-bottom culture dishes (NEST, China) and infected with *S. aureus* (MOI 200) for 6 h. The cells were washed three times with DPBS and treated with gentamicin (50 μg/mL) for 1 h. After treatment, the cells were rinsed three times with PBST and incubated with ice-cold 4% formaldehyde in PBS for 15 min at RT. To visualize the cytosol, the cells were permeabilized with 5% Triton X-100 in PBS for 10 min and then rinsed with PBST for 5 min. Samples were blocked using 1% BSA in PBST for 1 h and then incubated overnight at 4°C with anti-vimentin guinea pig polyclonal antibody (GP53, PROGEN, Heidelberg, Germany) and anti-*Staphylococcus aureus* antibody (ab20920, Abcam). The samples were then reacted with Alexa 488-conjugated anti-guinea pig secondary antibody (ab150185) and Alexa 594-conjugated anti-rabbit secondary antibody (ab150080, Abcam) in the dark for 2 h. A washing step with 0.1% Tween 20 in PBS was performed between each step. To stain the nucleus, the cells were incubated with 0.5 μg/mL DAPI (D9594, Sigma Aldrich) for 1 min. All plates were scanned at 100x magnification using a Leica SP8 CLSM (Leica Biosystems, Vista, CA, USA) at Sungkyunkwan University (Gyeonggi-do, Korea).

### 4.8 Flow cytometry

*S. aureus*-infected HaCaT cells were fixed with 4% formaldehyde at RT for 30 min. After cooling for 5 min, cells were centrifuged at 14,000 rpm for 5 min at 4°C. The supernatants were removed, and the cells were washed 3 times with PBS. 500 μl of ice-cold 0.1% Triton X-100 was added to each tube and incubated at RT for 30 min. The cells were then centrifuged at 14,000 rpm for 5 min at 4°C and washed three times. Following this, 500 μl of blocking buffer was added to each tube, briefly vortexed, and incubated on ice for 30 min. The supernatants were removed by centrifugation and aspiration. The cells were treated with vimentin monoclonal antibody (MA5-16409, Invitrogen) and incubated overnight at 4°C. After washing three times with PBS, the cells were treated with goat anti-rabbit IgG H&L (Alexa Fluor 488) (ab150077, Abcam) for 1 h and then washed with PBS. Flow cytometry analysis was performed at the Gyeonggido Business & Science Accelerator (GBSA, Gyeonggi-do, Korea) using a FACSAria II cell sorter (BD).

### 4.9 Fluorescence activated cell sorting (FACS) analysis

HaCaT cells were infected with *S. aureus* for the indicated times and subsequently fixed using 4% formaldehyde at 37°C for 20 min. After cooling for 5 min, the cells were incubated with an anti-vimentin antibody (diluted 1:200 in PBS) at RT for 1 h. Following a wash with 0.5 mL of ice-cold PBS, the cells were treated with anti-mouse IgG Alexa Fluor^®^ 568 (diluted 1:1000; Abcam, ab202504) for 30 min. After resuspending the cells in 0.5 mL of PBS, flow cytometry analysis was conducted at the Gyeonggido Business & Science Accelerator (GBSA, Suwon, Korea) using a FACSAria II cell sorter (BD).

### 4.10 Cell viability assay

HaCaT cells were seeded at 60% confluence in 96-well white/clear plates and cultured overnight. The cells were treated with *S. aureus* prepared at a concentration of 10^8^ CFU/mL in DPBS solution and incubated for the indicated times. EZ-Cytox reagent (Daeil Lab Service, Seoul, Korea) was added to each well and incubated for 30 min. Absorbance was measured at a wavelength of 590 nm using an EL800 microplate reader (Biophotometer, Eppendorf, Hamburg, Germany).

### 4.11 Data analysis

Significant differences in means between the groups were assessed using one-way analysis of variance (ANOVA), followed by Tukey’s honestly significant difference (HSD) *post hoc* test, or two-way ANOVA. The data presented represent the mean ± standard deviation (SD) from triplicate experiments. Differences were considered statistically significant when the p-value was less than 0.05.

## Data Availability

The original contributions presented in the study are included in the article/**Supplementary Material**. Further inquiries can be directed to the corresponding authors.
