# Supplementary material for: Staphylococcus aureus utilizes vimentin to internalize human keratinocytes
Source: Front Cell Infect Microbiol. 2025 Feb 21;15:1543186. doi: 10.3389/fcimb.2025.1543186 (PMC11885264; doi:10.3389/fcimb.2025.1543186)
Supplement: Supplementary file 1 [file DataSheet1.pdf]

1. Supplementary materials and methods

1.1 Manufacture of recombinant EsxB protein

Staphylococcus aureus Ess extracellular protein B (EsxB) gene was amplified by polymerase chain reaction (PCR) from the total genomic DNA of *S. aureus* (ATCC 29523). The forward primer used for the EsxB amplification was 5'-AAC ATA TGG GTG GAT ATA AAG GGA TT-3', while the reverse primer was 5'-AAC TCG AGT CAT GGG TTC ACC CTA TC-3'. Following cloning into the pET28a vector using the restriction enzymes *Nde*I and *Xho*I, the EsxB recombinant protein was produced through isopropyl β-D-1-thiogalactopyranoside (IPTG) induction and purified using a His-tag purification system.

1.2. Quant sequencing

HaCaT cells were seeded onto 6-well plates at a density of 1 x 10^6 cells per well. The cells were subsequently infected with *S. aureus* at a multiplicity of infection (MOI) of 200 and treated with 100 μg/mL of recombinant EsxB for 6 h. Total RNA was extracted from the HaCaT cells using Trizol reagent, in accordance with the manufacturer's protocol (Invitrogen, Breda, Netherlands). The integrity of the RNA was assessed by visualization on a 1% agarose gel under UV light, and the RNA concentration was measured using a NanoDrop spectrophotometer (Thermo, Breda, Netherlands). Quantitative mRNA sequencing analysis was performed at Ebiogen (Seoul, Korea).

1.3. Reverse Transcription-Quantitative Polymerase Chain Reaction (RT-qPCR)

HaCaT cells infected with *S. aureus* were washed three times with Dulbecco's phosphate-buffered saline (DPBS) and resuspended in 1 mL of Trizol reagent (Invitrogen, Waltham, MA, USA) for total RNA extraction. Complementary DNA (cDNA) was synthesized from the total RNA using an iScript cDNA synthesis kit (Bio-Rad, Hercules, CA, USA). To quantify mRNA expression levels, RT-qPCR was performed using a CFX Connect real-time PCR detection system (Bio-Rad), and the PCR products were detected with SYBR Premix Ex II (TaKaRa, Shiga, Japan). The following sequences for forward and reverse primer pairs were used in this study: 5'-ACCGAGTTCAAGAACACCCG-3' and 5'-TAGTTGGCGAAGCGGTCATT-3' for vimentin, and 5'-AAGGTCGGAGTCAACGGATT-3' and 5'-GCAGTGAGGGTCTCTCTCCT-3' for glyceraldehyde-3-phosphate dehydrogenase (GAPDH). mRNA levels were normalized to GAPDH.

1.4. Bacterial Culture

Lactiplantibacillus plantarum K8 (KCTC10887BP) and *Enterococcus faecium* (DSM20477) were cultured in MRS broth, while *S. aureus* (ATCC 29523) was cultured in BHI medium for 16 h. The bacteria were harvested by centrifugation at 8000 x g and washed three times with DPBS, then resuspended in DMEM to achieve the desired concentration.

1.5. HaCaT cell internalization assay

HaCaT cells were treated with the indicated doses of Gram-positive bacteria and cultured in a CO_2 incubator for 6 h. The culture supernatant was collected from the cells, and 100 μL was spread

onto MRS or BHI agar plates to quantify extracellular bacterial colony-forming units (CFU). The HaCaT cells, from which the culture supernatant had been removed, were washed with DPBS, lysed with a hypertonic buffer, and then spread onto agar plates to determine the intracellular CFU of *S. aureus*.

1.6. Western blotting assay

Epithelial cell lines, including HT-29, HCT-116, and CT-26, were maintained in DMEM supplemented with 10% heat-inactivated FBS, 2 mM L-glutamine, 100 U/mL penicillin, and 100 μg/mL streptomycin. Cells were seeded onto a 6-well plate and incubated overnight at 37°C in a humidified incubator with 5% CO_2. The cells were stimulated with 1 x 10^8 CFU/mL of *S. aureus* for 6 h, after which the culture media were removed. Following two washes with DPBS, the cells were lysed with 2x Laemmli buffer and boiled at 100°C for 5 min. Cell lysates were subjected to 12% SDS-PAGE for the separation of protein bands. Proteins were transferred onto a PVDF membrane for 1 h a t 10 V. The membrane was blocked in blocking buffer for 1 h a t room temperature (RT). Subsequently, the membrane was incubated with an anti-vimentin antibody for 2 h at RT. After washing with TBST, the membrane was incubated with an HRP-conjugated anti-rabbit secondary antibody for 1 h a t RT. Following three washes with TBST, the protein bands were detected using ECL Select™ Western Blotting Detection Reagent and exposed on X-ray film.

2. Supplementary Figures

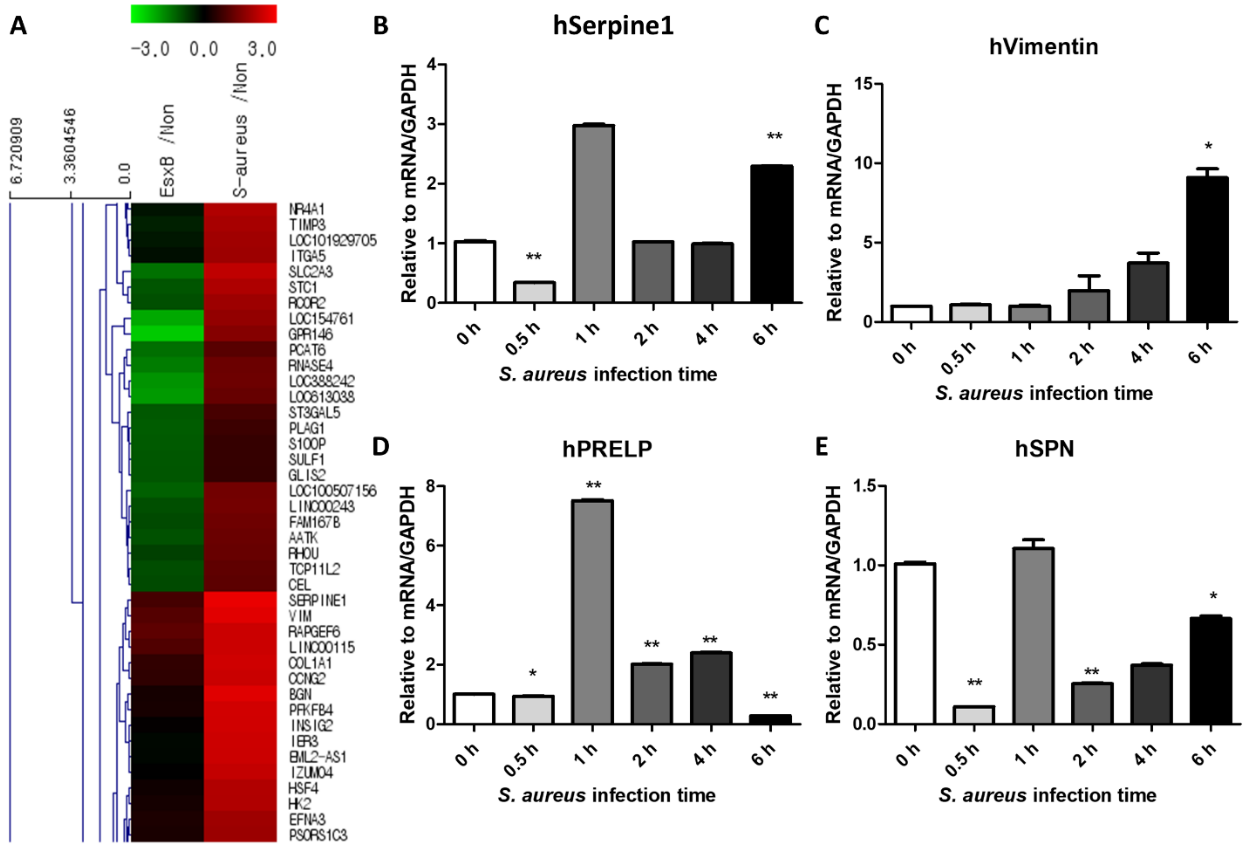

2.1 Supplementary Figure 1. Transcriptome analysis revealed that *S. aureus* infection increased vimentin expression. (A) HaCaT cells were treated with *S. aureus* and EsxB for 6 h, after which total RNA was extracted. The results of the transcriptome analysis were visualized using heatmaps. (B to E) Four main genes that exhibited increased expression in the transcriptome analysis were further validated by RT-qPCR. HaCaT cells were treated with *S. aureus* for the indicated times, total RNA was extracted, and cDNA was synthesized. RT-qPCR was conducted to assess the expression levels of four genes, including Serpine1 (B), Vimentin (C), PRELP (D), and SPN (E).

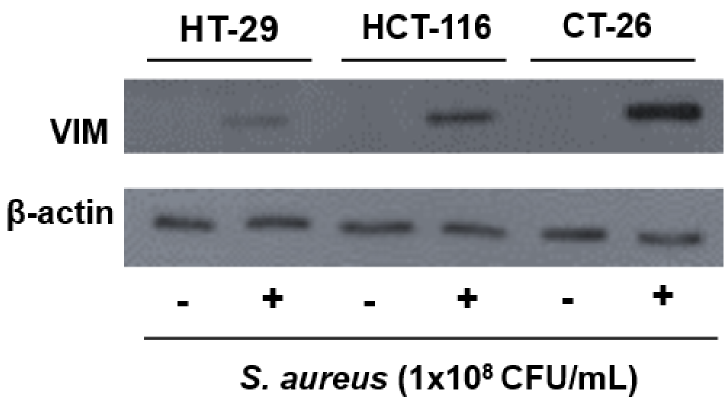

2.2 Supplementary Figure 2. The expression of vimentin in human and mouse epithelial cell lines was investigated. The study included HT-29 and HCT-116, which are human colorectal adenocarcinoma cell lines, as well as CT-26, a murine colorectal carcinoma cell line derived from BALB/c mice. These cell lines were treated with *S. aureus* for 6 h, and vimentin expression was analyzed using Western blotting (WB).

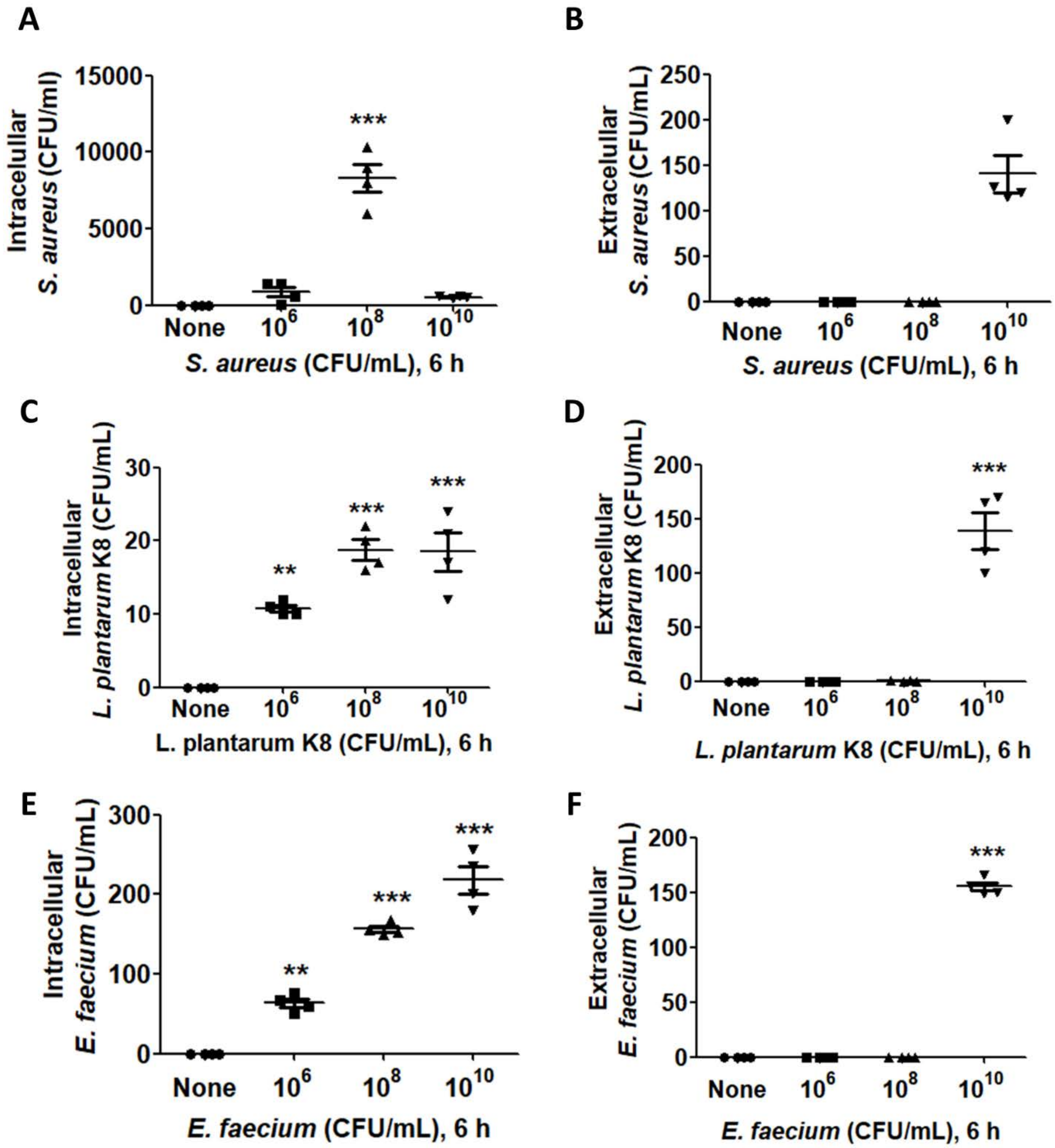

2.3 Supplementary Figure 3. Comparison of intra- and extracellular infection of pathogenic and probiotic Gram-positive bacteria. HaCaT cells were treated with the indicated doses of *S. aureus*, *L. plantarum*, and *E. faecium* for 6 h. Cell lysates and culture supernatants were plated on agar, and colony-forming units (CFUs) were counted. (A) Intracellular CFUs of *S. aureus*. (B)

Extracellular CFUs of *S. aureus*. (C) Intracellular CFUs of *L. plantarum*. (D) Extracellular CFUs of *L. plantarum*. (E) Intracellular CFUs of *E. faecium*. (F) Extracellular CFUs of *E. faecium*.

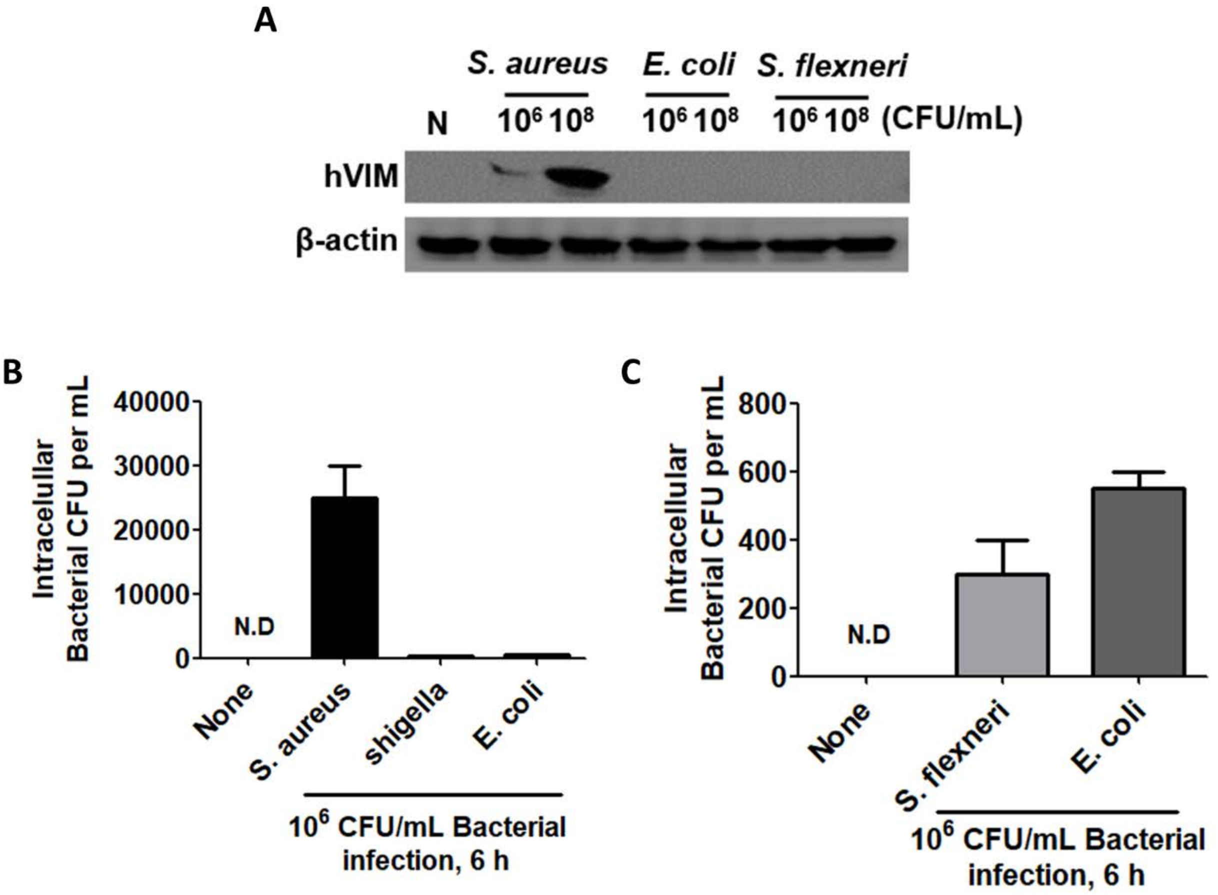

2.4 Supplementary Figure 4. Comparison of intracellular infection of *S. aureus* and Gram-Negative Bacteria. HaCaT cells were treated with the indicated dose of *S. aureus*, *E. coli*, and *S. flexneri* for 6 h. (A) Vimentin expression was analyzed using Western blotting (WB). (B to C) Cell lysates were plated on agar, and CFUs were counted. (B) Intracellular CFU of *S. aureus*, *E. coli*, and *S. flexneri*. (C) Intracellular CFU of *S. flexneri* and *E. coli*.
